# Continuing professional development barriers and recommendations: Perspectives of audiologists

**DOI:** 10.4102/sajcd.v71i1.1048

**Published:** 2024-08-20

**Authors:** Suvishka Barath, Andrew J. Ross

**Affiliations:** 1 Department of Family Medicine, College of Health Sciences, University of KwaZulu-Natal, Durban, South Africa

**Keywords:** continuing professional development, young audiologists, education, healthcare, private sector, barriers, recommendations, South Africa

## Abstract

**Background:**

Continuing professional development (CPD), a compulsory requirement of the Health Professions Council of South Africa (HPCSA), is undertaken by healthcare professionals (HCPs), including audiologists, to remain up-to-date with the latest developments, technology and best practices within their discipline. However, the low compliance rates of audiologists engaging in CPD need to be investigated to establish the barriers that audiologists encounter as well as possible interventions to improve their participation.

**Objectives:**

This study aimed to explore the barriers that audiologists encounter when participating in CPD activities and to highlight their suggestions for improving its uptake.

**Method:**

The descriptive qualitative research design entailed the use of semi-structured online interviews with 11 audiologists practising within the private sector in the province of KwaZulu-Natal, South Africa, their responses being thematically analysed.

**Results:**

Three barriers were identified, namely: (1) personal, (2) financial and (3) structural barriers, with eight subthemes and nine recommendations provided by participants.

**Conclusion:**

It is anticipated that implementing the proposed strategies will address the barriers and allow active engagement of audiologists in their continued education.

**Contribution:**

Limited literature has been documented on the barriers that young, private sector audiologists encounter within the South African context while also providing suggestions to address these barriers.

## Introduction

Continuing professional development (CPD) is a systemic learning approach pursued by healthcare professionals (HCPs) to maintain competency after completing their initial training (Giri et al., 2012). It is important for all HCPs, including audiologists, to uphold the highest standards of care, and remain current and competitive in an ever-evolving field. The intention being to update and enhance their knowledge, skills and experience, based on the evolving scope of practice to the most recent scientific findings. Effective CPD planning starts with a health education needs assessment, which can be obtained by assessing the perspectives of HCPs (Giri et al., 2012). Without a reasonable understanding of past education, knowledge gaps and the needs of the health workforce, it is unlikely that CPD activities will address their deficiencies in competency, and will only satisfy regulatory obligations (Giri et al., 2012).

Within the private sector, where audiologists operate in diverse clinical settings, ranging from independent practices to corporate healthcare environments, the imperative for CPD is particularly pronounced. Unlike their counterparts in academic or research institutions, private-sector audiologists often face unique challenges, such as time constraints, financial considerations, and the need to stay competitive in a rapidly changing market (Merry et al., 2023). In this context, CPD emerges not merely as a professional obligation, but as a strategic imperative for staying ahead in a dynamic and competitive field (Shamim & Rasheed, 2021).

Audiologists are categorised by the Health Professions Council of South Africa (HPCSA) under the Speech, Language and Hearing board (SLH), and as of 31 January 2024, the overall compliance rate for all professionals registered under this category was 37.2% (HPCSA, 2024). Audiologists are required to pay annual membership fees to the HPCSA and are encouraged to join professional associations such as the South African Speech-Language-Hearing Association (SASLHA) and the South African Association of Audiologists (SAAA), which provides CPD webinars and journal articles to members.

For audiologists to be licensed as independent practitioners working in South Africa (SA), irrespective of their employment status, they must comply with the CPD requirements of the statutory body, known as the Health Professional Council of South Africa (A Health Professional Council of South Africa, 2021). Currently, the HPCSA requires practitioners to obtain continuing education units (CEUs), which refer to the value of a learning activity for CPD (HPCSA, 2021). However, merely accumulating these points does not necessarily indicate genuine learning or improvements in the quality of the HCPs’ performance (HPCSA, 2021). This is because CEUs often measure participation in educational activities rather than the actual comprehension and application of the knowledge gained. Audiologists are currently required to obtain a minimum of 30 clinical CEUs, and five ethics, human rights or health laws CEUs (HPCSA, 2021).

The proposed HPCSA CPD guidelines will require HCPs to develop a learning plan based on their own needs assessment, which will address gaps in their knowledge and skills, and outline how the learning activity will influence patient outcomes (HPCSA, n.d.). In addition, the proposed guidelines have introduced new CPD requirements, which include topics related to professionalism, quality and safety, and communication. The categories of ethics and professional competency, which are required for the HCPs to maintain their registration annually, have been retained in the proposed guidelines (HPCSA, n.d.). The proposed guidelines will require audiologists to acquire a minimum of 30 annual credits in the following categories: (1) 25 points for professional competency; (2) 3 points for ethics and professionalism; (3) 1 point for safety and quality, and (4) 1 point for communication (HPCSA, n.d.). The guidelines are under review, and it is anticipated that they will come into effect sometime in the near future.

A review of the literature showed a lack of research on audiologists’ participation in CPD activities in the private sector in SA, a low-middle-income developing nation (World Health Organization, 2023). Given the poor compliance rates and paucity of research in this field, it is important to identify the barriers preventing their participation, as well as potential recommendations to address the challenges identified and improve their participation, engagement, and compliance with CPD activities.

Understanding the barriers to HCPs’ participation is essential in low- and middle-income countries such as SA, where non-financial constraints, such as human resource shortages, could influence the practicality of implementing the proposed guidelines (Van Rensburg, 2014). Furthermore, there is a need to examine both the context and the delivery of CPD programmes at a private sector level to identify effective solutions that could strengthen the participation of HCPs in CPD activities and ensure that they result in improved practices. However, very little is known about the factors that prevent audiologists from participating in such events, making it difficult to know how to address them. The aim of this study was therefore to explore the barriers that prevent audiologists from participating in CPD activities and to highlight their suggestions for improving the provision of CPD.

## Research methods and design

A descriptive, qualitative research design was adopted and entailed interviewing audiologists working in the private sector in KwaZulu-Natal (KZN) province between November 2023 and December 2023. This study reports on the barriers and recommendations encountered by private sector audiologists, building on the authors’ previous publication titled *Conceptualising the Experiences of Continuing Professional Development of Young Private Sector Audiologists as an Attribute of Andragogy* (Barath & Ross, 2024).

### Study population and sampling strategy

Participants consisted of audiologists from the private sector and were recruited via a poster that was distributed across various social media platforms that are popular within the audiology community in KZN. Audiologists who were interested in participating directly contacted the researcher. Purposive sampling was followed by a snowball technique, where the initial respondents were asked to provide the names of other eligible participants who could be contacted. Upon receiving a positive response, potential participants were sent an information document via email or WhatsApp informing them about the nature of the study to enable them to make an informed decision before consenting to participate. Those meeting the inclusion criteria and who were willing to participate were given informed consent forms. The inclusion criteria included audiologists who graduated between 2017 and 2022 because of the significant disruption caused by the coronavirus disease (COVID-19) pandemic on healthcare practices, including audiology. This period saw unprecedented challenges such as lockdowns, restricted patient access, and shifts towards telehealth services, which particularly affected early-career professionals who were still solidifying and strengthening their clinical skills and establishing their careers. Participants had to also be registered with the HPCSA as independent practioners and working in KZN’s private sector. Those with dual qualifications in audiology and speech-language therapy were excluded to prevent potential confounding of the results, as well as those working in both the public and private sectors.

### Data collection

Online interviews using WhatsApp voice calls were used and recorded with permission from the participant for later transcription and thematic analysis. The interviews were conducted in English, as all the participants were fluent speakers, the opening statement being ‘Tell me about your experiences with CPD and why you participate in CPD activities’. The semi-structured interview guide was based on issues identified in the literature related to barriers identified elsewhere and contained predetermined open-ended questions that covered issues related to their participation in CPD programmes. The interviews lasted between 20 min and 40 min.

The audio-recorded interviews were transcribed verbatim and analysed using NVivo, a qualitative data analysis program using deductive thematic analysis, the six-step analytical process identified by Braun and Clarke (2006). The data analysis was ongoing during the data collection process and after 11 interviews, no new themes emerged from the data.

A pilot study with two participants was conducted to assess the credibility of the data collection tool. An independent qualitative coder objectively analysed the data, which increased its trustworthiness and decreased bias. After independently identifying codes, categories and themes, a consensus meeting was held between the researcher and the independent coder at which they agreed on codes and themes.

### Ethical considerations

To ensure anonymity, each participant was allocated a unique code, depending on their sex, being categorised as either ‘F’ for female or ‘M’ for male with a number for consistency. The Humanities and Social Sciences Research Ethics Committee at the University of KwaZulu-Natal (HSSREC) provided ethical clearance (reference number HSSREC/00006281/2023). The participants’ personal names and information related to their private practices were redacted, ensuring privacy and confidentiality.

## Results

The participants’ details are followed by the three themes identified, those being personal, financial and structural barriers.

### Description of participants

The ages of the participants (eight females and three males) ranged from 23 years to 26 years, their duration of working in the private sector being 1–5 years, the average and the median number of years being two (Table 1). The most common CPD activities were online and reading journal articles.

**TABLE 1:** Participants’ description.

Participant code	Gender	Age (years)	Years worked in private practice	Type of CPD activity
P1	F	23	1	Online journal articles In-person workshop
P2	M	23	1	Online journal articles Online training courses Webinars
P3	F	24	1	Online journal articles Webinars
P4	F	23	1	Online journal articles Webinars
P5	F	25	2	Online journal articles In-person courses Webinars
P6	F	25	2	Online journal articles
P7	M	26	2	Online journal articles In-person workshops
P8	M	26	2	Online journal articles Online training courses Webinars
P9	F	26	5	In-person workshops Online journal articles Online training courses
P10	F	24	2	Online journal articles In-person workshops
P11	F	23	1	Online journal articles

CPD, continuing professional development; F, female; M, Male.

Table 2 shows the three themes that affected their participation in CPD activities, with their associated eight subthemes and nine recommendations.

**TABLE 2:** Themes, subthemes and recommendations.

Barriers to participating in CPD		Recommendations to overcome the barriers
Themes	Sub-themes	
1. Personal barriers	1.1. A lack of motivation	1. Undertake a needs analysis
	1.2. A lack of relevant CPD topics	2. Provide clinically relevant CPD activities
		3. Improve CPD monitoring
		4. Improve accessibility
2. Financial barriers	2.1. Cost of CPD activities	1. Ensure affordability
	2.2. Location of CPD activities	2. Aligning pricing to the CPD value
	2.3. Professional Association Body fees	3. Provide in-person skills training
	2.4. Network connectivity	
3. Structural barrier	3.1. A lack of information on CPD activities	1. Improve notification system
	3.2. CPD activities during working hours	2. Equal distribution of clinical and ethical CPD points

CPD, continuing professional development

### Theme 1: Personal barriers

Personal barriers refer to the attitudes of HCPs towards CPD, as well as the self-perception of themselves as learners who need to engage with such activities.

#### Sub-theme 1.1: A lack of motivation

A number of participants expressed negative attitudes towards engaging in CPD and a lack of personal motivation because of the courses not being of interest to them. For some, the only reason to participate and adhere to the CPD requirements was their statutory bodies mandate, as they found none of the available courses applicable to their needs:

‘Honestly, just because HPCSA stipulates that, so I just do it for the points.’ (P9, F, 26, In-person workshops)

#### Sub-theme 1.2: A lack of relevant continuing professional development topics

Other participants indicated that although they do attend the CPD activities and it does help maintain their knowledge, the CPD content is sometimes irrelevant, and they are not able to apply it to their clinical practice for a variety of reasons. At times, this is because of the lack of equipment as expressed by Participant 9:

‘So like DPOAEs [*Distortion Otoacoustic Product Emissions*] we won’t get to do it because we also don’t have the equipment for that right now. So DPOAEs even though I’ve attended CPDs and stuff, it’s almost useless unless I get a referral from another audiologist and then I get those results.’ (P9, F, 26, In-person workshops)

### Recommendations to overcome the personal barriers

#### Recommendation 1: Undertake a needs analysis

Most participants recognised the need for CPD activities and suggested conducting a needs analysis to accurately assess their training requirements. This would motivate them to engage more frequently in CPD activities, as the relevant content could significantly enhance their clinical practice.

‘Maybe you can have a needs analysis that is sent out to all the audiologists in the province, and maybe see from there which areas people are interested in, then we can give points according to which subject is important, like the most important. What I’m saying is that we can strategize on our own, so we still making up the points but it’s actually making a difference towards our careers, our profession.’ (P7, M, 26, Online journal articles)

#### Recommendation 2: Provide clinically relevant continuing professional development activities

Participants highlighted that it is important for the planners and organisers of CPD activities to consider their areas of practice and the relevance it has within their facility. This would allow for the CPD activities to suit the requirements of audiologists as seen in the participants’ response:

‘They should really put out more activities that are relevant especially in private practices. We need more courses that focus on diagnostics and hearing aids. For example, let’s say you are working at a private practice that usually focuses on adult populations and focuses on your traditional air conduction hearing aids. However if you take a look at the CPD activity being put out, most focus on cochlear implants and how to program cochlear implants and all the rehabilitation on cochlear implant. Now that isn’t very relevant to what you are doing, I think they should just put out more relevant CPD activates.’ (P2, M, 23, Online journal articles)

#### Recommendation 3: Improve continuing professional development monitoring

Participants acknowledged that they often read journal articles to obtain points to remain compliant with the HPCSA. However, the activities that they attend are at times irrelevant to their scope of practice. They therefore suggest that HPCSA take more time to monitor the type of CPD activities that professionals engage in:

‘I feel HPCSA has to have strict ways of monitoring whether the person is engaging or doing the CPDs that’s under their profession, that would really help because it’s pointless to pay large amounts to only find out we doing the articles but it’s not under our profession so that’s not really helping us in terms of growing our knowledge. So HPCSA needs to be stricter if the main reason for HPCSA making us to do CPD is to have much better knowledge within our professions, they need to implement stricter rules.’ (P3, F, 24, Online journal articles)

#### Recommendation 4: Improve accessibility

As a result of the lack of access to relevant articles, participants find themselves completing irrelevant activities so as to be adherent to the requirements of the statutory body. They, therefore, suggested that the HPCSA / Speech and Language Board / professional associations provide a platform that can provide updates on clinically relevant CPD activities:

‘More easy access, specifically for audiology, because some of the articles I’ve been doing are like general articles like medicine articles just to get points. So more articles specific to audiology and also a nice platform or some way of getting these courses shown like if there’s a course running in Durban or somewhere just easy access to be able to see where they occurring and when is the course. More accessibility to Audiology articles to be able to easily do it, do the questionnaire and submit it and get the points.’ (P5, F, 25, Online journal articles)

Participants suggested that the platforms that provide CPD activities should be widely publicised to assist audiologists in gaining access to these activities:

‘I think this is information that we as professionals, are supposed to have. You know you shouldn’t ask yourself now, how should I go about trying to get CPD points you should know. I know that this is the route that I need to take in order to ensure that I get my points for doing the work.’ (P7, M, 26, Online journal articles)

‘I think this is something that should even be on the HPCSA website or something like that. In addition, they should be sending us emails notifying us that you can get CPD points via those methods.’ (P7, M, 26, Online journal articles)

### Theme 2: Financial barriers

Financial barriers are factors that negatively affected participants’ participation in CPD activities.

#### Sub-theme 2.1: Cost of continuing professional development activities

Participants indicated that they were demotivated from engaging in reading journal articles that were CPD-accredited and attending CPD-accredited activities because of the exorbitant prices as there was often a high cost for accessing one CPD article as expressed by the participant:

‘It might be that sometimes courses are a bit too expensive. It might be going to the thousands of rands. So, it’s something that you definitely have to budget once and set aside.’ (P10, F, 24, Online journal articles)

Although all participants were working in private practice, they recognised that many young audiologists were unemployed after completing their community service because of the budget constraints of the Department of Health, which prevented them from being absorbed into the public health system. At this early stage of their careers, they did not have the experience to open their own private practice, nor were there many opportunities for employment for those who had such limited experience. This resulted in many encountering financial constraints that not only affected their ability to pay their annual registration fees but also to engage in the CPD activities for which fees were required:

‘HPCSA doesn’t consider the people that are not working, they just say you have to have 30 CPD points for this year.’ (P8, M, 26, Online journal articles)

#### Sub-theme 2.2: Location of continuing professional development activities

Participants stated that CPDs obtained at conferences situated at distant locations may result in them incurring high costs, which can prohibit them from attending:

‘And that’s the thing - those things are only held once a year and it’s not always accessible to everyone because it’s either not in your province or it’s quite costly when you have to travel for certain congresses, yeah, meetings. You’ve got to book accommodation and flights and everything. So those once-a-year options are sometimes difficult for certain people.’ (P6, F, 25, Online journal articles)

#### Sub-theme 2.3: Professional association body fees

While participants acknowledged that joining SASHLA involved a cost but provided CPD accredited articles to members at no additional charge, they did not receive free activites from HPCSA, even though HPCSA is the body responsible for monitoring their CEU’s. The requirement to pay registration fees for two professional bodies was onerous and questionable, as it added to their costs:

‘In fact, it’s also quite expensive if you look at it, if you pay for those specific professional bodies and then you have to pay for your HPCSA annual fees. It’s quite expensive.’ (P6, F, 25, Online journal articles)

#### Sub-theme 2.4: Network connectivity

Participants highlighted that the expense of data as well as network connectivity was a barrier for some audiologists to access CPD materials and attend online courses. The network issues, which were worse in some areas affected their ability to participate in online activities and caused frustration, which impacted on their desire to adhere to the CPD requirements:

‘I’d say data, so the CPD articles are already expensive, but also we need to have data for us to access them. So now these are two things at once we have to have. Money to access them and data and considering that our areas sometimes don’t have networks and we are hindered in accessing them in terms of data which we don’t have every time. So as much as I want to be compliant with my CPDs, now the network is the problem.’ (P3, F, 24, Online journal articles)

### Recommendation to overcome the personal barriers

#### Recommendation 1: Ensure affordability

Participants suggested that the CPD activities should be more cost-effective and affordable:

‘So if I can say something can be changed, maybe they can try not to make them more expensive.’ (P8, M, 26, Online journal articles)

Furthermore, participants suggest that cognisance be taken into account of professionals who are unemployed and provide assistance for these professionals to remain compliant with CPD requirements as expressed by the participant:

‘I think providing free courses for people that are not working.’ (P11, F, 23, Online journal articles)

#### Recommendation 2: Aligning pricing to the continuing professional development value

While participants acknowledged that the CPD activities are quite costly, their suggestion was for the allocation of points to increase to justify the monetary value of the CPD activity as seen in the participants’ statement:

‘They need to increase the points for the courses if it’s expensive. They can’t say the cost is R600 and the points they provide are three points.’ (P11, F, 23, Online journal articles)

#### Recommendation 3: Provide in-person skills training

Participants indicated that it would be extremely beneficial to their clinical practice if CPD activities were practical and in-person while in keeping with the needs of audiologists. This will also assist in eliminating the challenge of exorbitant fees for network connectivity that many participants encountered:

‘So, I think if they had to change something it would be more in-person, practical CPD activities that we could physically use because for a lot of it is just online articles. And I think skills training would be so much better. I see there’s actually wax removals, for example, wax removals are supposed to be done by audiologists, but a lot of them don’t do it and they say it’s because they don’t have enough experience or enough training, and so there’s certain areas where audiologists can improve the care they provide and the services they provide if they’re given more training. So if they had to host workshops on things like this in person where you could physically go and you and other audiologists could practice certain things or physically be a part of, like, skills training.’ (P8, M, 26, Online journal articles)

### Theme 3: Structural barriers

Structural barriers refer to the practices that restrict audiologists’ access from engaging in CPD.

#### Sub-theme 3.1: A lack of information on continuing professional development activities

Most participants admitted that they lacked information regarding CPD activities that can contribute towards points:

‘I only know of the articles and the webinars and the in-person workshops. So, I’m not aware of any other ways to obtain these points. Well, they don’t make it public knowledge.’ (P6, F, 25, Online journal articles)

#### Sub-theme 3.2: Continuing professional development activities during working hours

Participants experienced inconvenience in their private practice when attending CPD activities as the CPD activities do not always align with their work schedule, with many occurring during working hours. This negatively affected their practice as patients have to be seen at a later stage to accommodate the audiologist’s adherence to CPD:

‘So especially if you are someone that’s working in the private sector it means you’re working five to seven days a . And you don’t always have the time to go out to workshops that are happening during the day or you’ve got to take leave from work.’ (P8, M, 26, Online journal articles)

‘Or it could be something that I have to align with my work schedule. I can’t book patients during that time that the workshop is being conducted.’ (P10, F, 24, Online journal articles)

For activities that took them away from their practice for more than a few hours, they had the challenge of finding a replacement while attending CPD courses to keep their business operating, as it was simply not possible to close it for a few days:

‘It’s more challenging now because for me I can’t just leave my practice, I’d have to find a locum. I can’t just leave the practice to attend these courses and stuff.’ (P5, F, 25, Online journal articles)

### Recommendations to overcome the structural barriers

#### Recommendation 1: Improve notification system

Participants suggest that HPCSA should reconsider its notification system and platform used to disseminate information as they indicated a lack of knowledge of the range of possible CPD activities, resulting in them being non-compliant. In addition to informing them of the range of options available to acquire CPD points, participants indicated that there should be regular updates from the HPCSA informing them of their CPD status (not only when they are non-compliant):

‘Notification needs to be better we don’t pay much attention to these CPD points up until now you are notified ‘Be careful you are not compliant’ because they usually send SMS or something so now you start panicking and do those things and then try to get more. So, I’ve encountered that problem before. I started to be serious about this when they sent me that.’ (P8, F, 26, Online journal articles)

#### Recommendation 2: Equal distribution of clinical and ethical continuing professional development points

The participants indicated that there should be more journal articles focusing on the ethical and legal aspects of private practices, as these aspects are not well covered at university. They recognised that their knowledge deficiencies in these areas meant that they were not prepared for private practice:

‘Transiting from community service to private practice is a little bit overwhelming. I think they should put out more CPD activities that focus on the ethics as well as the legal side of practice. For example, medical malpractice I honestly didn’t know what medical malpractice was, it’s so important to get medical malpractice insurance in private practice. So, if I was aware of that maybe by doing a CPD course that focused on the ethics and the legal side of private practice I would be more informed. Yes, I actually think there should be a balance between the ethical and clinical side because both are equally important.’ (P10, F, 24, Online journal articles)

## Discussion

Enhancing and promoting professional competence is the goal of CPD as the ultimate beneficiary will be the patient. However, as shown in the participants’ responses, there are several barriers to accessing CPD. This study may possibly be the first study to explore the barriers that young audiologists encounter as well as their perspectives on how to address these challenges, particularly within the private sector.

Participants in this study indicated a lack of motivation towards engaging in CPD. This is because of a failure to recognise the significance of engaging in ongoing education, poor applicability of the CPD content to their practice, and the costs associated with CPD activities. This lack of motivation leads to HCPs engaging in these activities simply to get the points and ‘be compliant’ rather than the desire to improve their practice. A research study that investigated the professional development of radiographers in KwaZulu-Natal found that when radiographers are able to identify their own specific CPD requirements, there is a change in attitude and motivation as well as greater participation in these activities (Mung’omba & Botha, 2017).

Andragogy has identified the need for CPD activities to be relevant to practice if HCPs are to actively participate. Participants indicated encountering numerous irrelevant CPD activities, which they attended simply to access the CPD points on offer. They highlighted the importance of CPD activities aligning with their scope of practice within their facility. In a discussion document, the HPCSA suggested that an individual needs assessment followed by a learning plan should be developed by every HCP based on their own needs (HPCSA, n.d). Implementing such a learning plan would ensure that CPD activities are relevant to the HCP. The research findings of this study concur with published finding, which highlights the importance of HCP actively applying theory to practice, which then results in more effective learning (Harden & Laidlaw, 2020).

Participants recommended re-examining the monitoring of CPD activities as often the CPD activities do not signify true learning or a shift in their clinical performance. This need for monitoring has been recognised by the Ministry of Health in Ethiopia where a dedicated case team in charge of monitoring of CPD activities of HCPs has been established (Merry et al., 2023). This concept can be applied in SA through the implementation of a ‘2-stage process’. The first stage would involve screening and then accrediting the proposed CPD activities, and thereafter the second stage will involve reviewing submissions by the HCP to determine whether the activity led to improved patient care. The stricter monitoring of CPD activities among medical, nursing, and midwifery cadres in sub-Saharan Africa was shown to improve the application of learning to practice (Feldacker et al., 2017b). Such tracking of CPD participation could be used as a quality assurance tool to ensure that the CPD activity translates into best practice (Feldacker et al., 2017a). Although HPCSA has a tracking system to notify practitioners of their compliance status, it does not address the quality of the CPD activites nor does it ensure effective knowledge acquisition that improves patient care.

Many participants encountered financial constraints while attempting to engage in CPD activities, with the cost as a significant barrier to participating. While online activities potentially make CPD activities more affordable, the cost of data, subscriptions to professional body as well as the cost of some courses are significant barriers. Online CPD activities may reduce the cost of transport and accommodation but they are not a panacea as data costs can be significant when listening live to a webinar, and connectivity is patchy in many parts of KZN. As a result of the challenge of network connectivity, participants often felt excluded from participating in CPD. This concurs with research findings in Tanzania where a lack of internet access and consistent mobile coverage negatively impacted HCPs’ ability to adhere to CPD requirements (Feldacker et al., 2017a).

Participants also suggested the need for more in-person skills training to allow for the transfer of acquired knowledge into best practices rather than online CPD events. Research has shown that using technology to offer online CPD does not adequately address the demands of clinical practice. This is because clinical practice demands not only knowledge-based learning but also skills-based learning (Berndt et al., 2017). In addition, participants highlighted the importance of meeting with other audiologists where practical skills can be taught during the CPD activities. In-person meetings provide young audiologists the opportunity to receive constructive feedback and evaluations from their colleagues. This feedback mechanism will help them to identify areas for improvement and refinement of practical skills (Khoza-Shangase et al., 2021).

Innovative solutions are needed to ensure that there is a balance between online CPD activities and in-person activities to ensure that audiologists can acquire skills and knowledge they need to provide quality care. Such solutions could include local journal clubs consisting of various private sector audiologists, which could play a crucial role in stimulating CPD as they will provide a structured platform for engaging with this research, critically appraising evidence-based practices, and fostering a culture of lifelong learning while networking with peers (Ilic & Maloney, 2014).

A lack of technical resources was identified by participants who lack the equipment to put their CPD knowledge into effective practice. While it is beneficial for private practices to consider having the relevant equipment available to allow audiologists to provide these services, it is important to recognise that not all practices may align with the diverse fields within the scope of practice in audiology. Therefore, this should be seen as an open suggestion for comprehensive practices, rather than a mandatory requirement as each practice offers different services based on patient needs. This concurs with the research findings that investigated the CPD of medical, nursing, and midwifery cadres in sub-Saharan Africa (Feldacker et al., 2017b).

The cost of CPD is not a unique barrier to SA and has been reported in other studies, such as in Sudan (Elshami et al., 2016). Therefore, there is a general need to ensure the affordability of CPD materials. The literature identifies a need for professional bodies to mobilise resources to develop and distribute appropriate training materials to advance its mission to develop a skilled workforce (Giri et al., 2012). This will provide a way to address the financial constraints that HCPs face when implementing CPD programmes, as experienced by participants in this study. In addition, funding or subsidies would enable young audiologists to participate and would support CPD organisers in planning, implementing and assessing CPD activities as a lack of funding is a major obstacle to running these programmes (Feldacker et al., 2017b).

A lack of knowledge of how to acquire CPD materials negatively affected participants’ engagement with CPD activities. This is consistent with the findings of a Zambian study, which found that the lack of learning activities was a barrier to the radiographers’ participation in their CPD (Mwansa, 2018). Furthermore, there was poor awareness by participants of the kind of activities that could contribute towards CPD points consistent with the research study findings of the study in Zambia. Participants suggested more active promotion and marketing of CPD activities by the HPCSA, and SASLHA might positively influence HCPs’ attitudes and increase their participation in CPD activities. The literature identifies a lack of information and poor communication or notification of CPD events negatively impacts HCPs’ compliance with CPD activities, which aligns with the research findings of this study (Naidoo & Naidoo, 2018). An increase in the awareness of the range of CPD activities that HCPs could participate in would also give them a range of cost options.

In addition, during the undergraduate training of audiologists, there should be an augmented focus on private practice and instructions on accessing CPD activities. This ensures that upon graduation, audiologists exhibit confidence in identifying CPD opportunities. Furthermore, the integration of a designated section within CPD platforms tailored for private practitioners is imperative. This would facilitate comprehensive readiness for audiologists entering private practice. Attending to site accessibility and user-friendliness is essential in reducing the existing gap in the HCPs’ preparedness and the provision of relevant CPD activities.

Participants in this research study highlighted that because of the nature of private practice, when engaging in CPD activities they are unable to book patients as there is only one audiologist in the practice. This aligns with research findings from a study conducted in sub-Saharan Africa where because of a lack of human resources, specifically locums to cover the clinical work, audiologists are often unable to access and/or participate in the CPD activities that they desire (Feldacker et al., 2017b). Participants indicated that their attendance to CPD may negatively affect the production levels of their practice. A study that looked at the CPD of HCPs in developing countries suggested that the amount of time audiologists spend away from their employment engaging with CPD activities can be reduced by using strategies such as on-the-job training and blended learning (Giri et al., 2012). However, this is dependent on operational needs at the facility where the audiologist works. This was not seen in any of the participant’s responses in this study.

As per the current HPCSA requirements, audiologists are expected to obtain a minimum of 30 clinical CEUs and 5 CEUs for ethics, human rights, or health laws (Health Professionals Council of South Africa, 2021). However, most CPD activities focus on clinical practice with little emphasis on the ethical issues in practice. Participants therefore suggested that there should be an equal emphasis on ethical and clinical issues as they found the ethical aspect to be of equal importance to the clinical aspect considering the nature of their private sector. Providers of CPD activities should not neglect the ethical training of HCPs and should provide more activities to improve the ethical knowledge of HCPs. This concurs with a research study investigating the CPD of medical, nursing and midwifery cadres in Malawi, Tanzania and SA (Feldacker et al., 2017a). It is also critical for HCPs to understand that although cost is a significant factor, CEU points need to be allocated according to the academic value of the activity and not the monetary value. Excessively expensive courses should be regulated by HPCSA as currently it is a free market and one can change what they want for the CPD activity.

### Limitations

A number of limitations may have affected the findings, these being that most of the participants had only worked in the private sector for no more than 2 years, which means that they had limited experience with CPD activities in that sector. Their responses may therefore not reflect those who had worked for many more years in the private sector and knew how to navigate the CPD system.

## Conclusion

The findings of this study highlight several significant barriers that private-sector audiologists face during their participation in CPD. Overcoming these barriers requires a multi-faceted approach. By addressing these barriers and promoting a culture of lifelong learning, private-sector audiologists can overcome challenges and stay current as well as adapt to the evolving landscape of their field to provide optimal care for their patients. Furthermore, audiologists should be involved in every stage of the CPD process, including system planning and design, as they are essential to the system’s ability to deliver health outcomes.
